# In-amplifier and cascaded mid-infrared supercontinuum sources with low noise through gain-induced soliton spectral alignment

**DOI:** 10.1038/s41598-020-65150-6

**Published:** 2020-05-19

**Authors:** Kyei Kwarkye, Mikkel Jensen, Rasmus D. Engelsholm, Manoj K. Dasa, Deepak Jain, Patrick Bowen, Peter M. Moselund, Christian R. Petersen, Ole Bang

**Affiliations:** 1 DTU Fotonik, Department of Photonics Engineering, Technical University of Denmark, 2800 Kgs. Lyngby, Denmark; 2 School of Physics, Sydney Nano Institute, University of Sydney, Sydney, 2006 NSW Australia; 3 NKT Photonics A/S, Blokken 84, 3460 Birkerød, Denmark; 4 NORBLIS IVS, Virumgade 35D, 2830 Virum, Denmark

**Keywords:** Nonlinear optics, Solitons, Supercontinuum generation, Optics and photonics, Physics

## Abstract

The pulse-to-pulse relative intensity noise (RIN) of near-infrared (near-IR) in-amplifier supercontinuum (SC) sources and mid-IR cascaded SC sources was experimentally and numerically investigated and shown to have significantly lowered noise due to the fundamental effect of gain-induced soliton-spectral alignment. The mid-IR SC source is based on a near-IR in-amplifier SC pumping a cascade of thulium-doped and ZBLAN fibers. We demonstrate that the active thulium-doped fiber not only extend the spectrum, but also to significantly reduce the RIN by up to 22% in the long wavelength region above 2 μm. Using numerical simulations, we demonstrate that the noise reduction is the result of an interplay between absorption-emission processes and nonlinear soliton dynamics leading to the soliton-spectral alignment. In the same way we show that the RIN of the near-IR in-amplifier SC source is already significantly reduced because the spectral broadening takes place in an active fiber that also introduces soliton-spectral alignment. We further show that the low noise properties are transferred to the subsequent fluoride SC, which has a RIN lower than 10% (5%) in a broad region from 1.1–3.6 *μ*m (1.4–3.0 μm). The demonstrated low noise significantly improves the applicability of these broadband sources for mid-IR imaging and spectroscopy.

## Introduction

Spatially coherent laser sources such as SC sources^1–9^, frequency combs^10,11^, and optical parametric oscillators (OPOs)^12^ covering the mid-IR spectral region have received considerable attention owing to the existence of the many molecular resonances within this region. These are vibrational fingerprints (fundamental and recurring overtones) that are used to characterize materials based on their unique absorption features^11,13^. The technology platforms to reach the mid-IR region include fibers^1–4,7,9^, integrated optical waveguides^10,12,14^ and bulk media^8^. Here, we focus on fiber-based SC sources because of their robustness and ability for power scaling to generate high power spectral density over the whole mid-IR spectral bandwidth. Recent development in near-infrared (near-IR) and mid-IR SC sources has led to applications within optical coherence tomography (OCT)^15–20^, hyperspectral microscopy^21–24^, absorption spectroscopy^25–27^ photoacoustic microscopy and spectroscopy^28–31^, making it an emerging platform for practical applications. For example, a state-of-the-art mid-IR OCT system operating at 4 *μ*m has been demonstrated, which uses a mid-IR SC source and an ultra-fast up-conversion detector with a 1 *μ*m bandwidth (3.58–4.63 *μ*m) to obtain a record axial resolution of 8.6 *μ*m (5.8 times better than the much slower system in^19^). With this mid-IR OCT system it is possible to obtain real-time images of subsurface features in highly scattering samples, which are not accessible to OCT systems operating at visible or near-IR wavelengths due to the reduced penetration depth^16^.

Accessing the mid-IR region using standard pump sources in the near-IR is a well-known technique, which involves cascaded spectral broadening in several nonlinear fibers able to transmit beyond the 2.4 *μ*m absorption edge of silica fibers^2–4,32^. Such a cascading scheme is constructed by mode field diameter (MFD) matching and connecting different fibers with progressively longer wavelength transmittance. Two key factors in selecting the fibers in an efficient cascade, is that the red edge of the pump SC must consist of solitons and that the zero-dispersion wavelength (ZDW) must be significantly shorter than the red edge of the pump SC^3,32,33^. In this case, sufficiently many of the solitons in the red part of the pump SC are able to also generate solitons (preferably higher-order solitons leading to fission) in the new fiber, which can then redshift further out in the mid-IR through soliton self-frequency shifting (SSFS)^34^. In most cascading configurations, one inevitably moves from the more robust silica fibers to the soft and fragile fluoride fibers. The damage threshold of these fibers is comparatively low and requires that an appropriate pulse energy is used. Consequently, the cascade is engineered such that the power is below the damage threshold at all stages.

An important parameter limiting the application of near-IR and mid-IR SC sources are the inherent spectral fluctuations, which are mostly a characteristic property of the nonlinear dynamics involved in the generation of the SC. Standard SC sources use long pump pulses (picosecond or longer) that break into a sea of solitons initiated by noise seeded modulation instability (MI), and thus the entirety of the broad spectrum suffers from high RIN^35–37^. There have been several attempts to mitigate this noise, such as undertapering^38^, seeding of the MI^39,40^ (limited low power^41^ and a phase-coherent seed^42^), and increasing the repetition rate to average over more pulses for an enhanced signal-to-noise-ratio (SNR)^20,43^. However, it has never been investigated what effect the SC cascading has on the noise. Naturally, the choice of fibers in the cascade will affect the nonlinear and linear (amplification) dynamics of the SC generation (SCG), which in turn will influence the noise properties.

There have been experimental studies of the noise dynamics in near-IR SC sources implementing different methods like the dispersive Fourier transform technique, where the spectral profile of an SC is transformed into a temporal delay profile that can be detected with a fast photodiode and an oscilloscope by sending it through a long length of highly dispersive fiber, whose dispersion is known in order to back-convert from time to wavelength,^36,44,45^. However, this requires a long length of highly dispersive fiber for stretching the pulses in time. This technique has the limitation of not being able to characterize the SC noise in the longer wavelength region due to the lack of suitable highly dispersive low-loss fibers. Wavelength resolved SC noise has also been studied using filters and photodiodes to capture the individual pulse-to-pulse statistics either with an electrical spectrum analyser (ESA) (to get the noise in dB/Hz)^37,46,47^ or with fast oscilloscopes^48–51^ but to date the technique has been limited to investigations in the near-IR. Studies of the average properties of mid-IR SC sources have been carried out with slow photodiodes in terms of the fluctuations in the total power^26^ and the polarization properties of the SNR^52^, but to date the RIN profile, i.e., the pulse-to-pulse spectrally resolved pulse energy variation as function of wavelength across the mid-IR SC spectrum, has remained an open question.

In this work, we experimentally and numerically investigate the RIN of a cascade-based mid-IR SC source comprised of three stages: an in-amplifier SCG stage, erbium ytterbium doped fiber amplifier (EYDFA), a power redistribution and red-shifting stage, thulium doped fiber (TDF), and a final red-shifting stage Z_*r*_F_4_-BaF_2_-LaF_3_-AlF_3_-NaF (ZBLAN). Our emphasis is on two fundamental effects: (1) In the EYDFA the gain band overlaps the MI gain bands and thus the solitons generated by the MI-induced pulse-breakup - What effect does that have on the solitons and the noise? (2) In the TDF in the second stage of the cascade, light is absorbed within the 1.6 *μ*m band and gain is provided in two bands at longer wavelengths above 1.8 *μ*m - what effect does this have on the solitons and the noise? We experimentally observe that the RIN is strongly reduced in the SC out of the EYDFA, compared to standard sources based on spectral broadening in a passive nonlinear fiber, and that the RIN at the longer wavelengths is further reduced after propagation in a short piece of TDF. We numerically investigate this phenomenon using pulse propagation simulations with the generalized nonlinear Schrödinger equation (GNLSE). Results at two pulse repetition rates (100 kHz and 200 kHz) are presented for the entire cascade to show how the RIN is affected in the final stage of the mid-IR spectrum.

## Results

### Measured noise profiles

**Figure 1:**
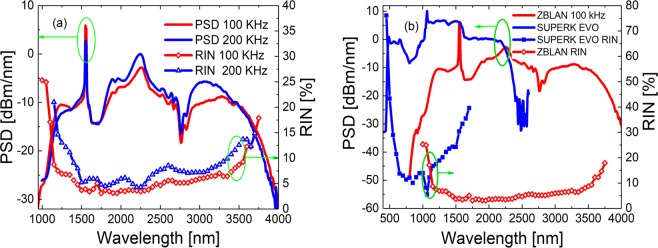
(**a**) Experimentally measured PSD (solid lines) and RIN (symbols) out of the ZBLAN fiber at 100 kHz (red) and 200 kHz (blue) repetition rates. (**b**) PSD and RIN profile of the 100 kHz configuration (red) and the corresponding values for an 20 MHz SuperK EVO SC source (blue), which is based on an ytterbium fiber laser pumping a silica photonic crystal fiber (NKT Photonics).

In Fig. 1(a), we show the measured PSD and RIN profiles of the mid-IR SC source, i.e., out of the ZBLAN fiber, for a repetition rate of 100 kHz (red) and 200 kHz (blue), for which the total output average power was 0.4 W and 0.7 W, respectively. The slightly higher total average power of the 200 kHz configuration is visible in the PSD profile. The RIN is seen to increase towards the spectral edges as in standard SC sources, and we see the 200 kHz configuration has a slightly higher noise than the 100 kHz configuration, which is explained by the fact that while the pulse duration of the seed laser is fixed, its peak power will drop when going to higher repetition rate^37^. However, the striking result is the RIN values, which are below 10% and 5% in an extremely broad bandwidth of 1.1–3.6 *μ*m and 1.4–3.0 *μ*m for the lowest noise 100 kHz configuration, respectively. Furthermore, the minimum RIN is seen to not be at the 1.55 *μ*m pump as in standard SC sources.

In Fig. 1(b) the PSD and RIN profiles of the lowest noise 100 kHz configuration (red) are compared to the corresponding profiles for a commercial 3.4 W, 20 MHz SuperK EVO industrial white light SC source (blue) based on an ytterbium fiber laser pumping a silica photonic crystal fiber (NKT Photonics). The RIN of the two sources have been measured with the same system using the same filter bandwidth, shown in Fig. 8, and can thus be directly compared. For the commercial source the RIN is above 5.5% at all wavelengths with a minimum of 5.5% at the 1064 nm pump. The low noise of the mid-IR source is very promising for its applications in spectroscopy and imaging.

The observed low noise is important. A first thing to consider is the influence of the filter bandwidth, where it seems feasible that a broader bandwidth would tend to reduce the noise. There is indeed a very small isolated decrease in the RIN of the 100 kHz spectrum at 2500 nm where the filter bandwidth jumps from 5 nm to 25 nm. However, this is not seen in the 200 kHz spectrum and in general the noise is increasing around 2500 nm, which seems to implicate that the filter bandwidths of 4.0–25.8 nm are in a regime where we can neglect the bandwidth influence for this particular source. Other nonlinear effects seem to have a dominating influence, as we will discuss in more detail below.

## Discussion

**Figure 2:**
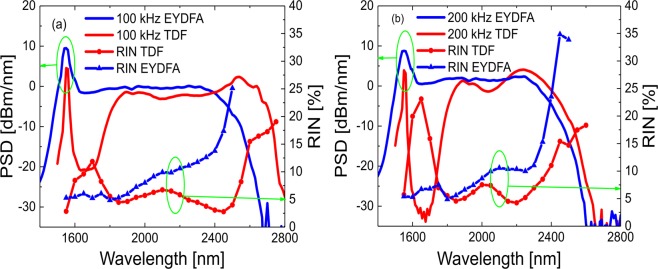
(**a**) Experimentally measured PSD (solid line) and RIN (line with symbols) out of the EYDFA (blue/triangle) and TDF (red/circle) at a repetition rate of (**a**) 100 kHz and (**b**) 200 kHz, respectively.

To understand the origin of the low noise, we investigate the evolution of the RIN profile along the cascade by measuring the PSD and RIN profiles of the SC out of the TDF and out of the EYDFA, focusing on the spectral region above the 1.55 *μ*m pump. The results are shown in Fig. 2 for the 100 kHz and 200 kHz repetition rates with 1.4 W and 1.8 W average power out of the EYDFA, respectively. When comparing the two repetition rates, it is important to note that the total continuum energy is larger at 100 kHz by a factor of 1.56 ((1.4W/100kHz)/(1.8W/200kHz)), which means that the soliton number is approximately a factor of 1.25 larger assuming that the fraction of residual pump and non-solitonic radiation is close to equal in both cases. Since the bandwidth of an MI-based SC obtained in a given fiber length increases with the soliton number, the spectrum is therefore expected to be broader at 100 kHz. This is also observed out of the EYDFA and even more clearly out of the TDF. The even stronger increased broadening out of the TDF at 100 kHz can be understood from the underlying amplification dynamics. Because the average output power of the EYDFA increases significantly from 100 kHz to 200 kHz using the same CW pump power, it is safe to assume that we are operating in the unsaturated regime where each pulse does not fully deplete the population inversion. Reducing the repetition rate gives the population inversion of the amplifier more time to recover in between pulses, resulting in higher per-pulse gain, but also increased amplified spontaneous emission (ASE) and parasitic gain^53^. In the TDF the dynamics are somewhat different because there is no CW pump to continuously provide population inversion. Instead, the gain originates from population inversion induced by absorption of the previous pulse, similar to the concept of tandem pumping^54^, eventually reaching a steady state after a number of pulses. Since the low repetition rate pulses carry more energy they also provide stronger amplification for the subsequent pulses, resulting in increased broadening in the TDF.

For both repetition rates, the RIN of the first SC out of the EYDFA increases from 5% around the pump to over 30% at the −20 dBm/nm (from the pump) edge around 2500 nm, as is standard for the soliton-dominated part of an MI-based SC above the pump^37^. The high RIN at the spectral edges of the SC is due to the low SNR and the appearance of rare events in the form of so-called rogue waves, which is a well-known phenomenon in SC generation^55^. In the central 1.5–2.3 *μ*m part of the spectrum the RIN remains between 5–10% despite the narrow ~ 5 nm bandwidth of the filters used in this regime (see Fig. 8). This is due to the lesser influence of SSFS variations close to the pump and the larger number of spectrally overlapping solitons.

Looking at the SC spectrum and RIN profiles out of the TDF, the first thing to notice is the spectral dip and corresponding peak in the RIN at 1.6–1.8 *μ*m. This is caused by the strong absorption of thulium within this window, which reduces the signal by 20–30 dB, thereby decreasing the SNR correspondingly. More interestingly, the introduction of the TDF is seen to strongly reduce the RIN for both repetition rates at wavelengths longer than 1.8 *μ*m, above the main 1.6–1.8 *μ*m absorption band of the TDF, where the 1.8–2.1 *μ*m main thulium gain band will amplify all solitons at those wavelengths, thereby causing them to increase their redshift due to SSFS. The second emission band at 2.2–2.5 *μ*m will further amplify the solitons that are either already present or redshifting into this band which will further push them towards the loss edge and thereby increase the red spectral edge of the SC, as is also observed.

The relative interplay of the two gain bands at 1.8–2.1 *μ*m and 2.2–2.5 *μ*m is non-trivial, as it is the cause of the low noise above 1.8 *μ*m. One could imagine that the second gain band would lead to a second dip in the RIN starting at around 2.2 *μ*m, because this is where it starts amplifying the redshifting soliton. This also fits for the RIN profile of the 100 kHz configuration, but not exactly for the 200 kHz configuration, where the second dip starts just above 2.0 *μ*m. The generally low RIN of the SC out of the EYDFA also corresponds with the presence of the Er gain band. This is supported by the fact that the RIN of the commercial SuperK source, which has a passive nonlinear fiber pumped by an ytterbium 1064 nm laser, has a significantly higher RIN, as seen in Fig. 1(b). To understand these issues we perform numerical modelling.

The soliton dynamics plays a vital role in the noise properties of an SC source, so to support our experimental findings and possibly explain the remaining questions a series of numerical simulations were performed. For simplicity, we focus on the 200 kHz configuration which has a low per-pulse gain and can therefore as a reasonable approximation be modelled as a uniform gain distributed evenly along the length of the TDF^53^.

 The spectral evolution in the first two nonlinear stages (EYDFA and TDF) of the cascade were simulated by solving the generalized nonlinear Schrödinger equation (GNLSE) using the fourth order Runge-Kutta integration scheme in the interaction picture with adaptive step size^38,56^. All simulations were performed with 2^19^ time sampling points and a temporal resolution of dt = 0.92 fs. The standard one-photon-per-mode with a random phase was added to the initial condition in the Fourier domain and a laser amplitude noise of 1% was added to the input amplitude^57^. Noise is only added to the amplitude because our pump laser is a directly modulated diode laser and not a mode-locked laser, in which case anti-correlated noise of the pulse length should also be added^57^. All PSD spectra are ensemble averaged over 72 simulations with random noise seeds and plotted using a running average of 10 nm to emulate the resolution of the Optical Scanning Spectrometer used in the experiments. All RIN calculations have been done using Gaussian-shaped 5 nm and 24 nm broad filters for wavelengths <2500 nm and ≥2500 nm, respectively, in accordance with the experimentally used filters characterized in Fig. 8.

**Figure 3:**
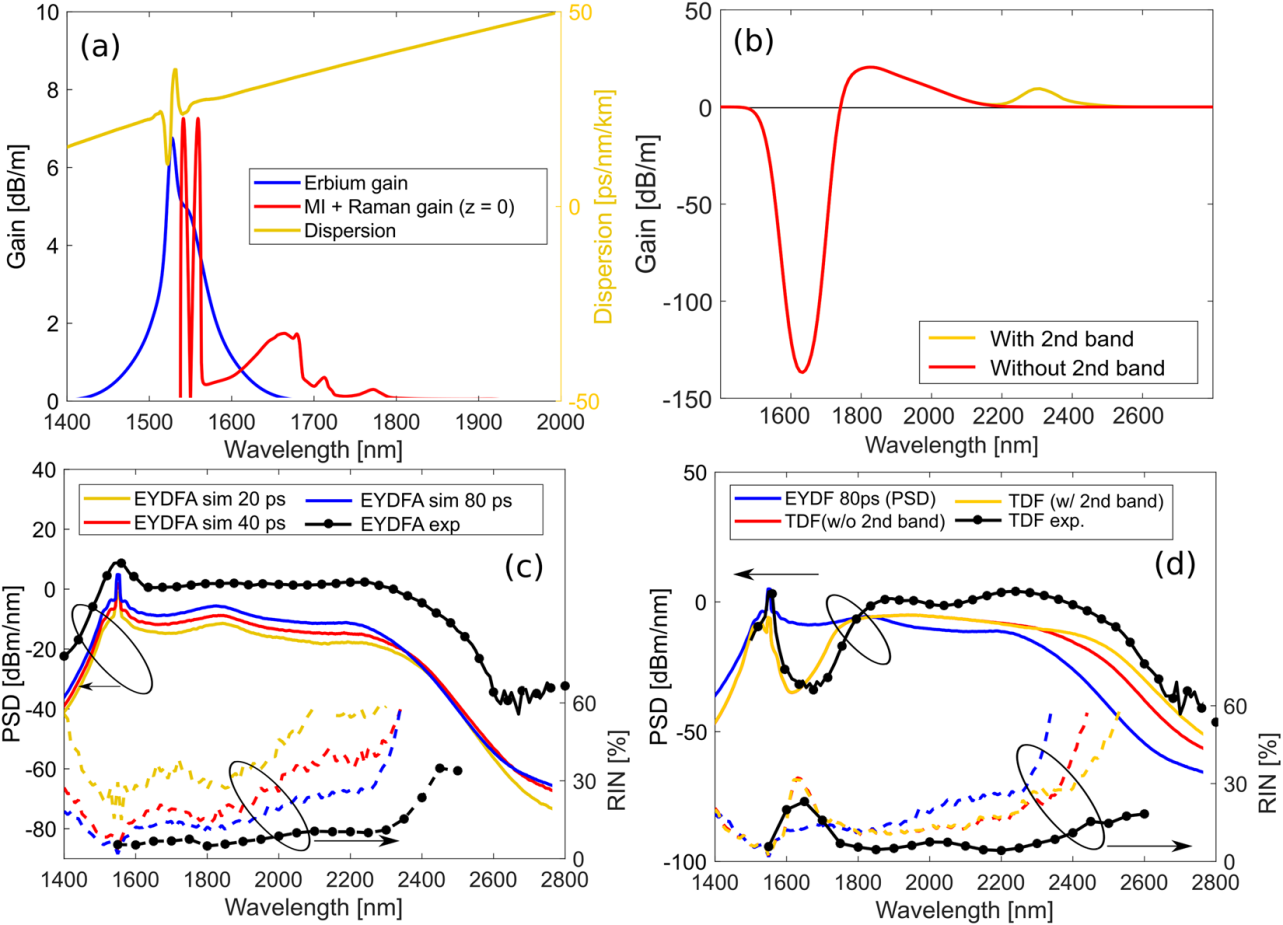
Results of experiments and simulation of EYDFA and TDF. (**a**) Erbium gain, MI +Raman gain and dispersion of the fiber. (**b**) TDF gain with and without the second band. (**c**) EYDFA output at varying pulse widths + comparison with experimental. (**d**) TDF output at 80 ps with and without the second band. Compared with EYDFA output (80ps) and experimental TDF output.

The initial pulse characteristics for simulating in-amplifier SC generation in the EYDFA was a Gaussian envelope with *P*_0_ = 470 mW peak power, chosen to match the experimental peak power of the 1 ns seed. In order to maintain high spectral resolution and reasonable computation time, the simulated pulse duration was reduced to 20, 40, and 80 ps. Despite the shorter pulse duration, the SC generation is still initiated by MI with the correct maximum MI gain ${g}_{MI}=\gamma$ ${P}_{0}$, where $\gamma =\mathrm{1.8(}Wkm{)}^{-1}$ is the nonlinearity of the EYDFA at the pump wavelength. The peak power rather than the pulse energy or soliton number was thus matched between experiment and simulation and 3 progressively doubled pulse lengths were used to study the trend when moving towards the nanosecond regime. Both the EYDF and the TDF are step-index silica fibers, so the dispersion of both fibers were calculated from the refractive index of pure silica and the NA, using the step-index eigenvalue equation^58^. The passive effect (i.e. outside the gain and absorption regions) of the dopants on the dispersion was neglected, although it is suggested by Thirstrup *et al*.^59^ that the addition of rare-earth dopants and other ion-dispersing dopants, such as aluminum, increases the refractive index. The effect of the emission and absorption processes are included as a gain and loss, respectively, that is constant along the length of the fiber. The effect of the 1.55 μm gain band on the dispersion was included via the Kramer-Kronig relations. The effect of all other absorption and gain bands on the dispersion is neglected. Furthermore, the EYDFA is backwards pumped so the 0.915 μm CW pumps were not included. The spectral shape of the Er gain profile is taken from^60^, and a peak gain of 6.7 dB/m was determined empirically by comparison of the simulated output spectrum with the experimentally observed spectrum. The EYDFA gain profile and its effect on the dispersion is shown in Fig. 3(a), together with the Raman and MI gain profiles. Importantly the Raman Stokes peak at ${\Lambda }_{Raman}=1663\,\text{nm}$ lies outside the Er gain band, whereas the MI Stokes peak at the input is at ${\Lambda }_{MI}=2\pi \text{c}/({\omega }_{0}-{\Omega }_{m})=1560\,\text{nm}$, where ${\Omega }_{m}={(2\gamma {P}_{0}/{\beta }_{2}|)}^{1/2}$

The TDF has no external pump, so here it is the output of the EYDFA with a peak around 1.55 *μ*m that excites the Tm atoms, which are then de-excited by primarily stimulated emission around 1.9 *μ*m. The TDF is 7% doped by weight and the peak core absorption in the 1.6 *μ*m region was empirically determined to be 137 dB/m, while the first gain band at 1.9 *μ*m was modelled with a peak gain of 20 dB/m^61^. The second gain band at 2.3 *μ*m was modelled with a gain of either 9 dB/m or 0 dB/m in order to investigate the effect of this gain on the soliton- and noise dynamics. The full gain/loss profile is shown in Fig. 3(b).

The results of the simulations of SC generation in 3.5 m EYDFA and 25 cm TDF are shown Fig. 3. From Fig. 3(c) we see that both the PSD and RIN profiles of the SC out of the 3.5 m EYDFA correctly approach the experimentally measured profiles, given by the black curves, as the pulse length of the pump is increased from 20 ps to 80 ps. The red edge of the PSD profile agree and the difference between 80 ps and 1 ns pulses of identical amplitude corresponds to approximately 11 dB in PSD, which is seen to correspond well with the experimental PSD level. This means that the 80 ps simulation can be trusted to qualitatively give the correct dynamics of the SC generation with the 1 ns pump.

The numerical results for the SC out of the TDF, shown in Fig. 3(d), also confirms the general experimental results, i.e., (1) the absorption region around 1.6–1.8 *μ*m has created a dip in the PSD and increase in the RIN, (2) the red spectral edge has been redshifted, and (3) the RIN out of the TDF is lower than the RIN out of the EYDFA above 1.8 *μ*m where the Tm gain bands start. Importantly we see that including the second Tm gain band at 2.3 *μ*m increases the spectral red edge by 50 nm and lowers the RIN above 2.3 *μ*m, thereby further improving the correspondence between the numerical and experimental results. This further underlines the conjecture that it is the gain bands and their amplification of the solitons and thereby increase in their SSFS induced redshift rate, which leads to the lowering of the RIN. A fundamental soliton will adiabatically reduce its pulse length while its amplitude is increasing due to the gain, such as to keep the soliton number fixed to 1, and it is this reduction in pulse length that is well-known to increase the redshift rate.

**Figure 4:**
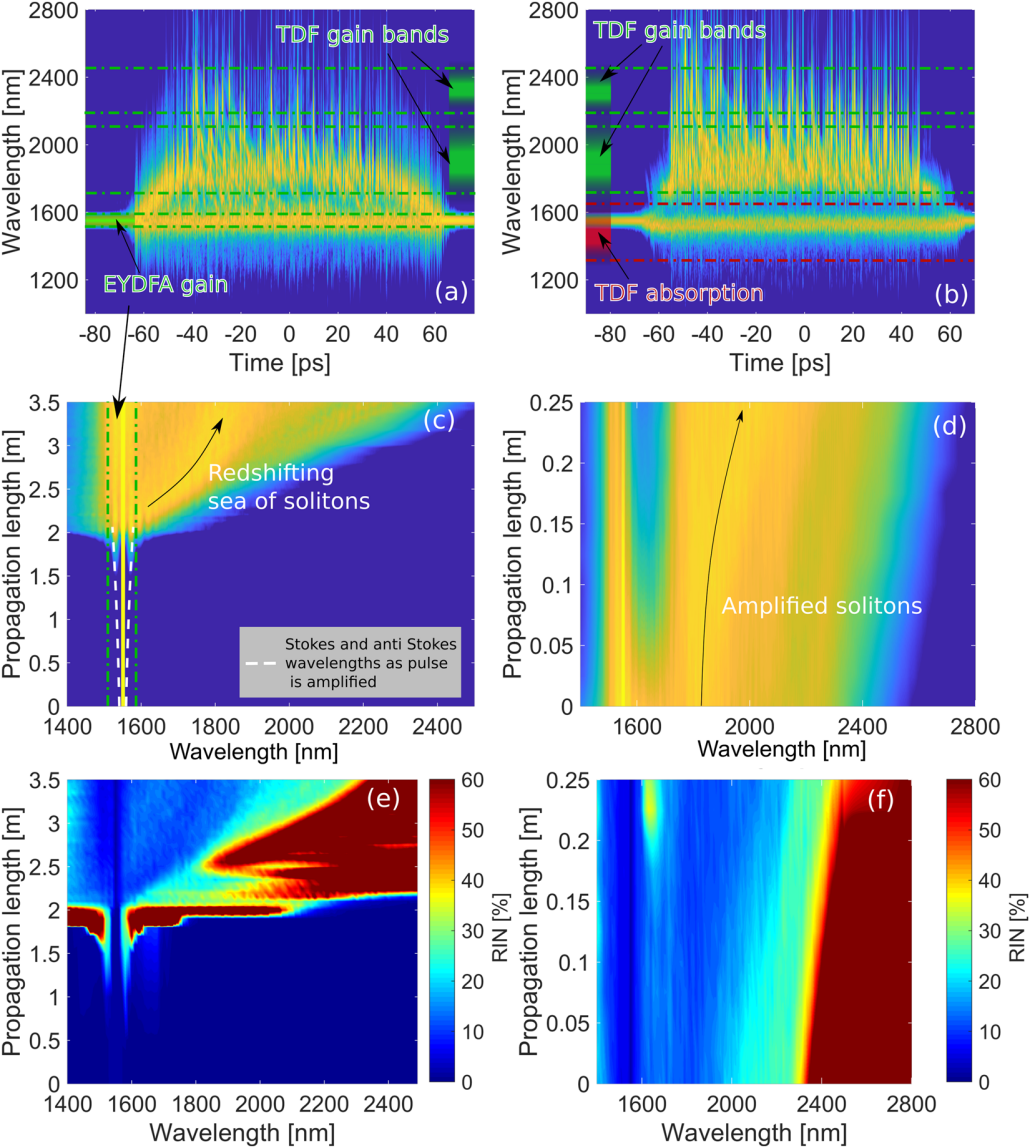
Results of experiments and simulation of EYDFA and TDF show. (**a**) the Spectrogram of EYDFA (**b**) the Spectrogram of TDF. (**c**): PSD evolution along EYDFA (**d**) PSD evolution along TDF. (**e**) RIN evolution along EYDFA. (**f**) RIN evolution along TDF.

Having confirmed the correspondence with experiments we now use the modelling to look deeper into the physics of the SC generation by showing the evolution of the PSD and RIN profiles along the fibers in Fig. 4(c–f), respectively. Spectrograms of the SC at the output of the fibers are shown in Fig. 4(a,b) with 3 dB/m gain and absorption bands marked in green and red, respectively, determined as the width at the absolute level of.

Let us first focus on the EYDFA. Due to the gain (5 dB/m at the pump) the pump power increases, which redshifts the MI stokes wavelength as seen by the white dashed line in Fig. 4(c). At 2 m, just before the pulse breaks up, the pump power has increased to P_0_ = 4.7 kW, which shifts the MI stokes wavelength to 1580 nm, still inside the Er gain band. Thus, the MI Stokes and anti-Stokes lines are always inside the broader Er gain band, which means that the Er gain band amplifies the MI process and all solitons generated in the MI gain region, thereby increasing their SSFS induced redshift rate. At the break-up length at 2m the 4.7 kW peak power 80 ps pump has a soliton number of N = 225. In a standard SC source, where MI generates a packet of solitons in a passive nonlinear fiber, these solitons will stay close to the pump for a long distance and slowly create larger solitons by energy transfer in multiple soliton collisions, that results in enhanced SSFS. In contrast, the packet of solitons here generated in the EYDFA will experience gain and thus be forced to redshift away from the pump much more rapidly. The red-shifting packet of solitons is marked by an arrow in Fig. 4(c). This will create a void at wavelengths just above the gain band. The spectral width will depend on the fiber length and dispersion, which is also clearly seen in the spectrogram in Fig. 4(a). The region of the packet of solitons will have high SNR and reduced RIN, which is also seen around 1.8 *μ*m in Figs. 3(c) and 4(e), both numerically and experimentally. This local dip in the noise is not just due to a high SNR but also due do a large degree of spectral alignment of the solitons imposed by the gain. This is an effect recently demonstrated in SC generation in under tapered fibers to give significant noise reduction due to the spectral alignment of the solitons imposed by the second ZDW moving into the SC in the taper^38^. This spectral alignment is clearly seen in the spectrogram in Fig. 4(a).

In the TDF the absorption band at 1.6–18 *μ*m will deplete the spectrum and the two gain bands in the 1.8–2.5 *μ*m region will even more strongly increase the redshift and impose spectral alignment of the solitons and thus lower the noise above 1.8 *μ*m. The spectral dip and the redshifting solitons are clearly seen in Fig. 4(d) and the strong spectral alignment is clearly seen in the spectrogram in Fig. 4(b). This convincingly explains the lowered noise above 1.8 *μ*m out of the TDF. The low noise is then finally transferred into the passive redshifting ZBLAN fiber, providing the measured low noise seen in Fig. 1.

## Methods

### Configuration of supercontinuum source

**Figure 5:**
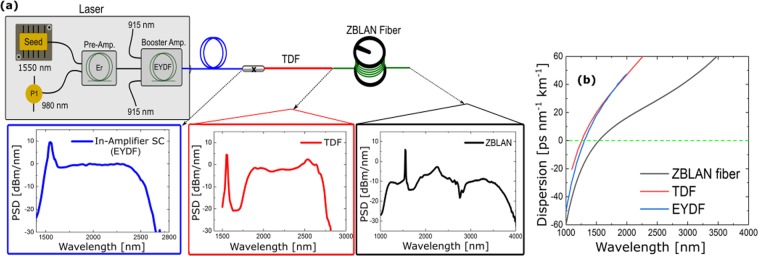
(**a**) Schematic diagram of the cascaded SC source with measured PSD profiles out of each stage shown in inserts. A 1.55 *μ*m seed laser with a few *μ*W of output power, variable repetition rate (10 kHz − 35 MHz), and ~1 ns pulse length is amplified to 120 mW in two stages. In the third stage an in-amplifier SC (1.5–2.4 *μ*m) is generated with up to 1.8 W of average power, which is then coupled into a TDF in order to shift the spectrum to ~2.8 *μ*m. This is further coupled into a ZBLAN fiber, which shifts the spectrum to around 4 *μ*m. (**b**) the dispersion of the fibers used in the cascade numerically computed in COMSOL.

The configuration for the SCG cascade is schematically shown in Fig. 5. The SC source is based on a MOPA architecture with a modulated (1.55 *μ*m) seed laser diode (pulse duration of ~1 ns, variable repetition rate from 10 kHz to 35 MHz) with three amplification stages. The first and second amplification stages are based on a few meters of double-clad Er doped polarization maintaining (PM) fiber pumped by a 980 nm laser diode. In the third amplification stage, an in-amplifier SC (1.5–2.4 *μ*m) is generated in an EYDFA (PM-EYDF −12/130-HE, Coherent Nufern) pumped by 915 nm pump diodes. The EYDFA is spliced to a short length of TDF, which redistributes the power absorbed by Tm-ions in the shorter wavelength region to the longer wavelength region. The single mode double-cladding TDF (DCF-TM-10/128, CoreActive) has a core and cladding NA of 0.22 and 0.45, respectively, with a clad absorption of 4.0 dB/m at 790 nm. This is subsequently spliced to a 15 cm long of mode-matching fiber for coupling to the ZBLAN fiber in the cascade. The mode-matching fiber is butt coupled to a 7.5 m long of ZBLAN fiber (ZSF − 7.0/125–265, FiberLabs), which extends the spectrum further out as shown in Fig. 5. At 100 kHz and 200 kHz repetition rates, the measured average power from the output of the EYDFA was 1.4 and 1.8 W, the output of the TDF was 0.9 and 1.2 W, and the output from the ZBLAN fiber was 0.4 and 0.7 W, respectively.

As explained above the ZDW of the fibers in the cascade is crucial. At all times, the ZDW of the fiber a supercontinuum is coupled into must be sufficiently below the red edge of the supercontinuum. Figure 5(b) shows that the first SC is generated in the EYDFA with a ZDW around 1.25 *μ*m (SC stage 1, identical to the third amplification stage), pumping at 1.55 *μ*m, well above the ZDW. A fairly flat spectrum to 2.4 *μ*m is generated in the EYDFA, which is limited by multi-phonon absorption in silica. This SC ultimately becomes the new seed for the TDF spliced to the EYDFA, which extends the continuum to approximately 2.8 *μ*m (SC stage 2) through the interplay of absorption-emission and nonlinear processes, which will be elaborated on shortly. The output of the TDF is spliced to a short piece of mode-matching fiber and then butt-coupled to a 7 *μ*m core ZBLAN fiber with a ZDW of 1.65 *μ*m, in which the broadening continues to around 4 *μ*m (SC stage 3)^3,4,32^, as shown in Fig. 5.

The absorption and gain profiles of the TDF is important for the efficiency of the SC cascade. First, the conventional cut-back method was used to measure the ground state absorption (GSA) at low power from the output spectra of 3 m, 1 m, and 0.5 m of TDF using the NKT SuperK compact (0.45–2.45 *μ*m, 22 kHz pulse repetition rate, average power of 80 mW) as shown in Fig. 2(a) (green dashed curve). From the absorption spectrum it is seen that the fiber exhibits narrow GSA lines at 0.46 *μ*m, 0.65 *μ*m, 0.79 *μ*m, and 1.2 *μ*m, and a broad GSA band at 1.6 *μ*m. The 1.6 *μ*m GSA band is a result of several closely spaced Stark components and their splitting into further sub-levels^60^. The observed GSA in the 1.38 *μ*m region can be attributed to aluminium ions used to increase Tm-ion dispersion to avoid clustering effects.

**Figure 6:**
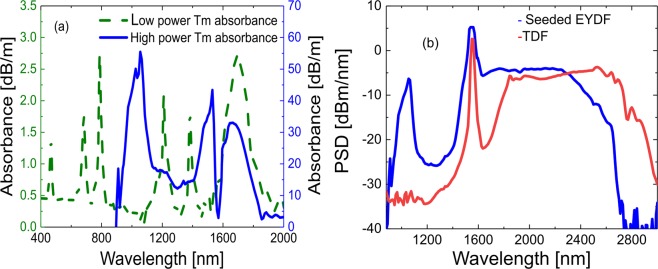
(**a**) Measured absorbance of the TDF using a low-power NKT SuperK compact SC source with the conventional cut-back technique (green dashed), and using the high power broadband EYDFA SC (solid blue). (**b**) Measured PSD out of the EYDFA (blue) and the TDF (red) at 10 kHz with 517 mW and 320 mW total average power, respectively.

**Figure 7:**
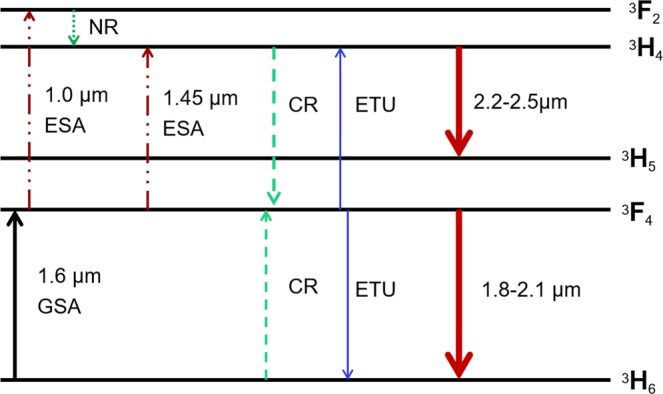
Energy level diagram depicting the possible absorption and emission processes in Tm ions under multiple excitations^60^ where the abbreviations ESA is excited stated absorption, GSA is ground state absorption, CR is cross relaxation, ETU is energy transfer up-conversion and NR is non-radiative relaxation.

To characterize the excited state absorption (ESA) bands of the TDF we used our own in-amplifier SC at 10 kHz in order to have enough power, which spanned from 1.4 *μ*m to 2.6 *μ*m, with a peak at 1 *μ*m due the ytterbium co-doping, as seen in Fig. 6(b) (blue curve). The absorbance measured with this high-power SC source is shown in Fig. 6(a) (blue curve), which shows an ESA band in the 1.45 *μ*m region, in agreement with previously reported TDF absorption measurements^62^. Another important ESA band is observed at 1.06 *μ*m, which coincides with the residual ytterbium peak. We have summarized the most relevant GSA and ESA lines in Fig. 7, together with the corresponding emission bands at around 1.8–2.1 *μ*m and 2.2–2.5 *μ*m. The GSA band around 1.6 *μ*m excites the Tm ions to the ^3^F_4_ manifold, which mainly feeds the first emission band at 1.8–2.1 *μ*m (from the ^3^F_4_ manifold to the ^3^H_6_ manifold). However, energy transfer up-conversion (ETU) between two Tm ions excited to the ^3^F_4_ manifold, through which they exchange energy non-radiatively to the ^3^H_4_ state and ^3^H_6_ state, as seen in Fig. 7, can also feed the second emission band at 2.2–2.5 *μ*m. This second band is also fed through ESA of excited Tm-ions in the ^3^F_4_ manifold, absorbing light within the 1.45 and 1 *μ*m wavelength regions, and transferring them to the ^3^H_4_ manifold (through non radiative transfer (NR) from the ^3^F_2_ manifold for the 1 *μ*m case). Finally cross relaxation (CR) processes, where excited Tm ions exchange energy with ground state ions, also contribute to the first and second emission bands.

**Figure 8:**
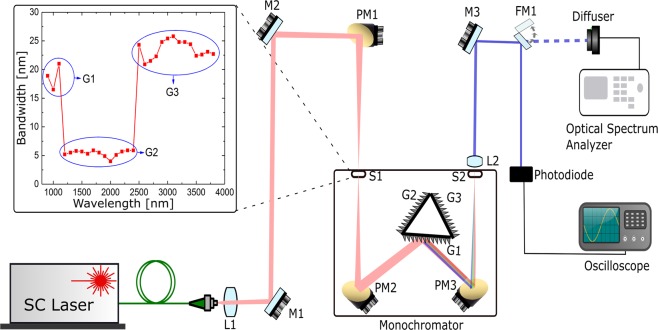
Experimental setup for investigating the relative intensity noise of the SC source over the bandwidth 1–4 *μ*m, a monochromator with appropriate gratings, a fast photodiode operating in the required wavelength region and a fast oscilloscope. Inset, Bandwidth of spectral filtering measured from the output of the ZBLAN.

### Noise measurement technique

In the following we will measure the RIN experimentally at the output of each individual fiber within the cascade separately, in order to get a deeper understanding of the noise properties of the SC at the different stages. The output beams were collimated using a CaF2 lens (L1), directed onto two protected silver-coated mirrors (M1 and M2), and then focused onto the input slit of a monochromator (SpectraPro2300i, Princeton Instruments) with a Czerny-Turner configuration using a 10 cm focal length off-axis parabolic mirror (PM1). The diverging beam from the input slit was re-focused using a parabolic mirror (PM2) onto one of three diffraction gratings mounted on a motorized rotation stage. The gratings G1, G2, and G3 had groove densities of 150, 600, and 150 grooves/mm at grating blaze wavelengths 0.8, 1.6, and 4 *μ*m, resulting in optimum wavelength ranges of 0.475–1.3 *μ*m, 1–2.4 *μ*m, and 2.4–6.0 *μ*m, respectively. After the gratings, the beam is focused onto the output slit (S2) by another parabolic mirror (PM3). The size of the output slit is used to adjust the bandwidth of the light. As such, there is a trade-off between the bandwidth and the strength of the signal measured by the detector. The diverging beam from the output slit is finally collimated with a CaF2 lens (L2). Using a flip mirror (FM1) the collimated beam can be directed either to a photodiode for noise measurements or to a diffuser connected by a ZBLAN patch cable to a scanning spectrometer for spectral characterization.

For the spectrally resolved noise measurements the SC was in this way filtered with a FWHM bandwidth of 4.0–25.8 nm FWHM in the region 0.9–3.8 *μ*m, where typically bandwidths of 10–30 nm have been used for near-IR RIN measurements in the 450–2310 nm range^37^. The spectral bandwidth of the beam exiting the monochromator is dependent on the sine angle of the incident light and the spacing between grooves of the diffraction grating and slit width. To avoid higher-order diffraction interfering with the measurements, a 1.8 *μ*m, 2.4 *μ*m long pass filter was placed in the setup before measuring longer wavelengths.

To cover the entire spectral range of the SC source two photodiodes were used. The first photodiode (PDA10PT-EC InAsSb Amplified detector, Thorlabs) has a variable bandwidth from 12.5 kHz to 1600 kHz, and an operating wavelength range of 1 *μ*m to 5.8 *μ*m with peak responsivity around 4.9 *μ*m. The second detector (Model 1811, New Focus) has a bandwidth of 125 MHz and an operating wavelength range of 0.9 *μ*m to 1.7 *μ*m with peak responsivity around 1.6 *μ*m. The photodiode voltage signal is recorded using a fast oscilloscope (HDO9404 4 GHz, Teledyne Lecroy) with 40 GS/s sampling rate and a varying bandwidth from around 1 GHz up to 4 GHz and up to 10 bits of resolution. Oscilloscope traces were recorded at a sampling rate of 1 GS/s, a bandwidth of 1 GHz, and a resolution of 9 bits, with a total of 10,000 acquired pulses. The RIN did not vary much about 0.05% from the value obtained using just 5,000 pulses and thus, 10,000 pulses were deemed statistically sufficient for RIN characterization. Assuming that the photodiode is fast enough (more than two times faster than the repetition rate according to the Nyquist criteria depending on the pulse shape) to follow the SC repetition rate, the pulse is much shorter than the photodiode response time, and that all the detected power levels are on the same linear part of its voltage versus power response curve, the voltage train recorded by the oscilloscope directly reflects the variation in power of the filtered SC. Before doing the pulse-to-pulse statistics on the maxima of the pulse train, we deduct a common average minimum in order to establish the noise floor for the measurement. The $\text{RIN}\,=\,\delta u\backslash \langle U\rangle$ is then finally calculated from the resulting series of measured maxima, where $\delta u$ is the standard deviation and $\langle U\rangle$ is the mean signal.

## Conclusion

To summarize, we have for the first time experimentally and numerically investigated the RIN for a Mid-IR cascaded SC source and demonstrated that it has a much lower RIN than conventional commercial SC sources. Specifically we show that the noise is lower than 10% (5%) in the broad region 1.1–3.6 *μ*m (1.4–3.0 *μ*m). Given that such a cascaded source is all about red-shifting solitons generated by noise-induced MI breaking up the pump, one would think that the noise would be high. Our experimental results and detailed theoretical investigation shows that this is not the case, and that in fact low noise is achieved because of the influence of the gain bands in the two active fibers in the cascade, the EYDFA and the TDF, which forces an increased spectral redshift and strong spectral alignment of the solitons. The fundamental process of low SC noise through soliton spectral alignment was just recently proposed in tapered fibers, in which it is the second ZDW that forces the solitons to align. Here we demonstrate that gain bands in the active fibers in a cascaded SC source have the same effect, which could aid the development of low-noise mid-IR sources in the future. Our studies further show, that any SC source based on in-amplifier SC generation, in which the gain band covers the MI gain bands leading to MI induced-generated solitons, will have a significantly reduced noise. This includes both in-amplifier SC sources based on ytterbium, erbium, and thulium doped fibers.

## Supplementary information

Supplementary information.
