# Supplementary material for: In-amplifier and cascaded mid-infrared supercontinuum sources with low noise through gain-induced soliton spectral alignment
Source: Sci Rep. 2020 May 19;10:8230. doi: 10.1038/s41598-020-65150-6 (PMC7237674; doi:10.1038/s41598-020-65150-6)
Supplement: Supplementary file 1 — Supplementary information. [file 41598_2020_65150_MOESM1_ESM.pdf]

In-amplifier and cascaded mid-infrared supercontinuum sources with low noise through gain-induced soliton spectral alignment

Kyei Kwarkye[1,+,*], Mikkel Jensen[1,+], Rasmus D. Engelsholm[3], Manoj K. Dasa[1], Deepak Jain[2], Patrick Bowen[3], Peter M. Moselund[3], Christian R. Petersen[1,4,+], and Ole Bang[1,3,4,+]

[1]DTU Fotonik, Department of Photonics Engineering, Technical University of Denmark, 2800 Kgs. Lyngby, Denmark
[2]School of Physics, Sydney Nano Institute, University of Sydney, 2006 NSW, Australia
[3]NKT Photonics A/S, Blokken 84, 3460 Birkerød, Denmark
[4]NORBLIS IVS, Virumgade 35D, 2830 Virum, Denmark
[*]corresponding. kkwar@fotonik.dtu.dk
[+]these authors contributed equally to this work

ABSTRACT

This document provides supplementary information on “In-amplifier and cascaded mid-infrared supercontinuum sources with low noise through gain-induced soliton spectral alignment”. Here we provide the detailed experimental and numerical investigations of a cascaded mid-infrared supercontinuum source. We investigate how the gain dynamics in the EYDF and TDF help in reducing the relative intensity noise in the final output of the fiber. We compare our experimental investigations to a commercial supercontinuum source and show that the performance of our source outweighs that of the commercial SC source.

Basis of the gain curves (EYDF and TDF) implemented in the simulation

With respect to the EYDF, we adjusted the gain such that the spectral broadening at the end of the fiber matches the experimental broadening. In addition, this turned out to agree quite well with the total gain (P_{output}/P_{input}), so we used this gain in the simulation. The shape of the gain was taken from the book Rare-earth-doped fiber lasers and amplifiers that is already referenced. For the TDF, the shapes were taken from[1]. The difference in shapes comes because the absorption and emission peaks overlap, so the blue part of the emission peak is "eaten" by the absorption as seen in Figure 4. The loss peak was adjusted such that the attenuation at 1600 nm matches the experiment and simulation, approximately 30 dB over the TDF. The first gain band was then roughly estimated to be around $1/3$ of the value of the absorption. Since we have little knowledge of the population dynamics (i.e. how much is stimulated emission from the same pulse, the next pulse, or spontaneous emission etc.), we deemed $1/3$ to be ok. That also gave a broadening similar to what we see for the 200 kHz at least.

Bandwidth of the monochromator

A monochromator with a 2f configuration was used to spectrally filter the broad supercontinuum source at each point in the cascade. The bandwidth of the filtered spectra was dependent on several factors. Such factors include focusing the beam at the exactly at the input focal point so that it exits exactly at the output focal point of the output slit. Another factor which determines the bandwidth of the filtered spectrum is the grating (sine angle of the diffracted beam) specification which includes the grating ruling which also has a dependence on wavelength. We could also control the bandwidth out of the monochromator m by adjusting the width of the output slit but this is a trade-off between how much power gets to the detector and the minimum bandwidth for each individually filtered spectrum for the broad spectrum. The slit width of the monochromator was kept at 2 mm apart. The spectrum was measured using a scanning spectrometer which operates in the desired wavelength region using Indium Flouride as a pick-up fiber. All spectra were measured with a scan step of 0.1 nm. The measured spectrum was normalised and the full width half maximum (FWHM) was computed for all wavelengths measured. Figure 1. shows the FWHM measured at 1600 nm.

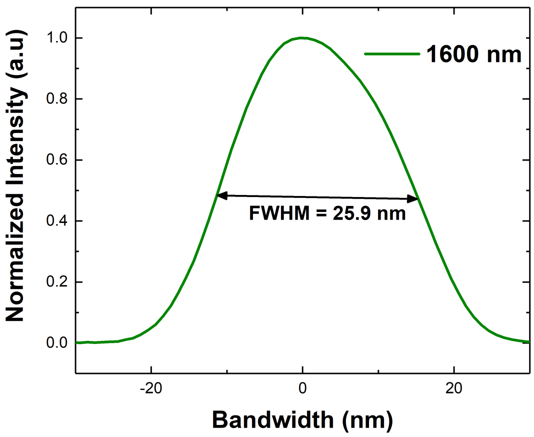

Figure 1. Bandwidth of the filtered spectrum at 1600 nm.

RIN change with respect to acquired pulses

To arrive at 10000 pulses that was deemed statistically viable with minimal error, we set the monochromator to a single wavelength. We then took pulses from 500 to 40000 and did statistical computation to see the variation in the relative intensity noise (RIN) to the number of pulses acquired. A plot of this is shown in Figure 2. It can be seen from the figure that after 10000 pulses the variation in the RIN was about 0.01% and that ch10000 pulses in the whole experiment was statistically sufficient.

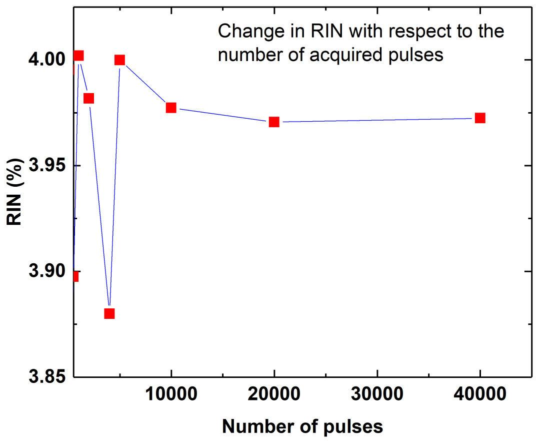

Figure 2. Plot of number of pulses with respect to RIN change.

Absorption curves of EYDF and TDF

In figure 3, we show the absorption curve of EYDF similarly used in our configuration. The figure is adapted from[2]. The maximum absorption is centered around 975 nm, but we used 915 nm pumps to probe this absorption leading to an emission at 1500 nm. There lies a small absorption centered around 1500 nm.

In figure 4, we also show the absorption curve of TDF adapted from[1]. We have also made a similar measurement with the specific TDF fiber used in our setup. The TDF show ground state absorption around 1600 nm and 1200 nm regions. There exist also an excited state absorption at the 1450 and 1000 nm regions. These excited state absorption can take place after the ground state absorption.

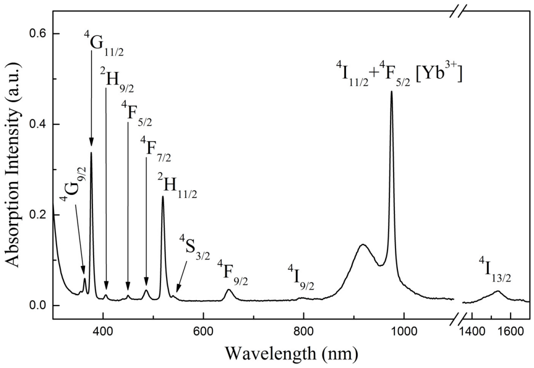

Figure 3. Measured absorption of EYDF. This figure was adapted from[2].

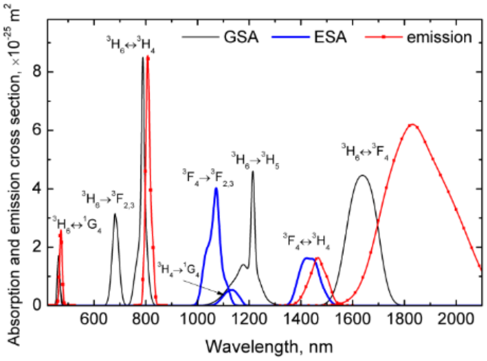

Figure 4. Measured absorption of TDF. This figure was adapted from[1].

References

1. Dussardier, B., Blanc, W., Peterka, P. *et al.* Tailoring of the local environment of active ions in rare-earth-and transition-metal-doped optical fibres, and potential applications. In *Selected Topics on Optical Fiber Technology*, 97–120 (InTech, 2012).

2. Zhang, W. *et al.* Optical properties of the yb/er co-doped silica glass prepared by laser sintering technology. *Opt. Mater. Express* **7**, 1708–1715 (2017).
